# Editorial: Advances in Understanding NeuroHIV Associated Changes in Neuroimmune Communication in the Combined Anti-retroviral Therapy (cART) Era

**DOI:** 10.3389/fneur.2021.763448

**Published:** 2021-10-05

**Authors:** Peter J. Gaskill, Jerel Adam Fields, Dianne T. Langford, Kelly L. Stauch, Dionna W. Williams

**Affiliations:** ^1^Department of Pharmacology and Physiology, Drexel University College of Medicine, Philadelphia, PA, United States; ^2^Department of Psychiatry, University of California San Diego, La Jolla, CA, United States; ^3^Department of Neuroscience, Lewis Katz School of Medicine, Philadelphia, PA, United States; ^4^Department of Neurological Sciences, University of Nebraska Medical Center, Omaha, NE, United States; ^5^Department of Molecular and Comparative Pathobiology, School of Medicine, Johns Hopkins University, Baltimore, MD, United States

**Keywords:** neuroAIDS, neuroimmunity, neuropathogenesis, neurotransmission, CART, neuroHIV

It has been almost 40 years since the first cases of AIDS were reported in Morbidity and Mortality Weekly in the fall of 1981 (1). During that time, almost 80 million people have been infected and more than 36 million people have died from HIV, with 1.5 million new infections and 680,000 deaths occurring in 2020. Despite these alarming numbers, there has been significant progress in controlling this pandemic as HIV infections and deaths have decreased 50–60% from their peaks in 1997 and 2004, respectively. This is largely due to the development and implementation of antiretroviral therapy (ART). In 2020, around two thirds of people living with HIV (PLWH) were treated with ART to suppress viral replication (2–6). Widespread access to ART has brought us to an inflection point in the pandemic, transforming HIV from a terminal diagnosis to a chronic condition.

Unfortunately, chronic infection and ART treatment have led to a variety of new health challenges, including long-term neurological dysfunction. In the absence of ART, PLWH showed rampant neuroinflammation and significant neuronal death associated with high rates of dementia. With ART, the neuropathology associated with HIV infection has become both more subtle and more complex. Importantly, even with suppression of viral replication to below the level of detection, neuropathological changes, minor neurocognitive dysfunction, depression, and other neuropsychiatric adverse events (NPAE) remain prevalent (5, 7–15), suggesting that the etiology of these conditions is not solely derived from viral replication. As Spagnolo-Allende and Gutierrez discuss in their review of cerebrovascular complications in neuroHIV, HIV proteins and associated neuroinflammation can initiate and/or exacerbate multiple types of vascular complications. Distinct phenotypes of vascular insults are associated with the presence or absence of ART in PLWH, so further study in this area is important given the growing incidence of cerebrovascular disease in PLWH on ART, particularly older individuals.

Neuronal health and function are maintained by complex, bidirectional interactions among neurons, astrocytes, microglia, and central nervous system (CNS) macrophages (16–18). Data suggest that neuroHIV stems, at least partially, from disruption of this communication, impairing neurotransmission, synapse formation/dissolution and neuroimmune communication. Some studies suggest that antiretroviral (ARV) drugs themselves may contribute to these changes in neuroimmune communication and neurological dysfunction (19–22). The data from Tripathi et al. support this possibility, showing that in both isolated rodent microglia and in HIV-1 transgenic rats, exposure to ART mediates microglial activation through oxidative stress-mediated lysosomal dysfunction. George et al., also demonstrate that antiretroviral drugs dolutegravir, emtricitabine, and efavirenz induce cell-type specific changes in ATP generation and mitochondrial respiration in epithelial and microglial cell lines, suggesting metabolic changes may contribute ARV toxicity in specific cell types. Dastgheyb et al. compared neurocognitive function between 929 virally suppressed women living with HIV and 717 HIV-uninfected women, identifying distinct neuropsychological profiles associated with both demographic and clinical variables, including the use of specific ARVs. Together, these data indicate more in-depth analyses of specific impacts of distinct ARVs both *in vitro* in discrete cell types and in humans in conjunction with analyses of a larger combination of variables could help to identify and/or predict neuropathological and neurocognitive response patterns to specific ART regimens.

Currently, the spread of HIV is increasingly driven by marginalized populations, including transgender women, sex workers, men who have sex with men and individuals using injection drugs. In each of these groups, the risk of HIV infection is 25 to 35 times higher than that in the general population (3). These populations have a high prevalence of substance use disorders (23–28) and hepatitis C (HCV) (29–32), so it is critical to evaluate their impact on neuroimmune communication, neuropathogenesis and neurological function. Paul et al. analyzed the neurocognitive response to ART in PLWH with co-occurring HCV infection, showing that both mono-HIV infected, and co-HIV/HCV infected individuals had significant neurocognitive improvement in response to ART. Notably, a subgroup of co-infected individuals with higher HIV plasma viral load and lower plasma CD4^+^ T cell count at baseline showed persistent motor deficits. In contrast, Matt et al. show that exposure to dopamine concentrations induced by the use of addictive drugs (33, 34) alters the efficacy of the CCR5 antagonist Maraviroc by increasing CCR5 expression on the macrophage surface. Cisneros et al., show that methamphetamine mediated activation of trace amino acid receptor 1 (TAAR1) triggers multiple signaling pathways in human astrocytes, regulating the expression of the glutamate receptor EAAT-2 through activation of CAMKII and Ca^2+^ release, and phosphorylating CREB via both the Ca^2+^ release and cAMP pathways. These data support substantial research indicating that both dopamine and substances of abuse can potentially exacerbate HIV infection and dysregulate associated cellular processes in the CNS.

While the above studies suggest that substances of abuse could exacerbate neuroHIV, other studies on abused substances suggest novel therapeutic activity for different drugs or neurotransmitter systems. In primary rodent neurons, League et al., show that blocking the activity of monoacylglyerol lipase (MAGL) reduces the neurotoxic effects of Tat. As MAGL drives metabolism of the cannabinoid receptor (CB_1_R) agonist, 2-arachidonoylglycerol, these data support the idea of using the endocannabinoid system as a target for neurotherapeutic adjuvants in ART-treated PLWH. Lin et al. show that the benzodiazepine, alprazolam (Xanax), alters the activity of the transcription factor RUNX1 and STAT5. Recent data show that benzodiazepines are overprescribed to PLWH and are associated with neurocognitive deficits, so these data suggest alprazolam may influence neuroinflammation and provide a mechanism underlying prior studies showing alprazolam can reactivate latent HIV. Han et al. use the iTat rodent model to show that Tat expression increases functional and structural indices of motor and sensory neuropathy, dysregulating the expression of proteins in the electron transport chain and the mitochondria. These effects were blocked by treatment with the muscarinic receptor 1 antagonist pirenzepine, which promotes mitochondrial biogenesis, suggesting the involvement of the muscarinic system in HIV distal sensory polyneuropathy. In another study using the iTat model, Joshi et al., compare acute vs. prolonged Tat induction and show prolonged Tat induction reduced locomotor activity and caused a small but significant increase in the ratio of MMP to TIMP1, while the acute induction of Tat reduced IL-6 mRNA expression.

The studies from this special collection reinforce that the development of neuroHIV is multi-factorial, driven by altered interactions among distinct types of CNS cells and interconnecting neurotransmitter, signaling and metabolic pathways. These data also collectively show that neuroHIV can be exacerbated by a variety of comorbidities and other factors including ART. Future studies should continue to evaluate the discrete impacts of ART and comorbidities, like substance abuse and HCV, as well as the combined effects of these conditions and their capacity to drive the growing number of complications associated with neuroHIV.

## Author Contributions

PJG, JAF, DTL, KLS, and DWW wrote the editorial and invited authors to participate in the collection. All authors contributed to the article and approved the submitted version.

## Funding

This work was supported by grants from the National Institutes of Drug Abuse (DA039005 and DA049227 to PJG, DA037830 to DTL, DA044838 and DA052859 to DWW), National Institutes of Mental Health (MH115819 to JAF, MH107340 to DTL, MH062261 pilot funding to KLS), National Institutes of Aging (AG066215 to JAF) and the W.W. Smith Charitable Trust (A2003 to PJG).

## Conflict of Interest

The authors declare that the research was conducted in the absence of any commercial or financial relationships that could be construed as a potential conflict of interest.

## Publisher's Note

All claims expressed in this article are solely those of the authors and do not necessarily represent those of their affiliated organizations, or those of the publisher, the editors and the reviewers. Any product that may be evaluated in this article, or claim that may be made by its manufacturer, is not guaranteed or endorsed by the publisher.
